# MethCancerDB – aberrant DNA methylation in human cancer

**DOI:** 10.1038/sj.bjc.6604219

**Published:** 2008-02-05

**Authors:** M Lauss, I Visne, A Weinhaeusel, K Vierlinger, C Noehammer, A Kriegner

**Affiliations:** 1 Austrian Research Centers GmbH – ARC, Molecular Diagnostics, Mendelstrasse 1, Seibersdorf A-2444, Austria

**Keywords:** DNA methylation, database

## Abstract

Early detection, classification and prognosis of human cancers by analysis of CpG methylation carry huge diagnostic potential. MethCancerDB collects and annotates genes and sequences from the abundance of published methylation studies and interlinks them to all methylation-relevant bioinformatical resources. MethCancerDB starts with 4720 entries from 348 sources and is freely accessible at http://www.methcancerdb.net.

Cytosine-5 DNA methylation epigenetically regulates gene activity in humans. Methylation occurs in CpG dinucleotides, which are relatively rare in the genome. Short stretches of DNA enriched in CpG nucleotides are termed CpG islands. About 88% of all human genes have such a CpG island in their promoter region (Kim *et al*, 2005). Hypomethylation is usually associated with gene activation and hypermethylation with gene repression or silencing. Aberrant DNA methylation at CpGs is crucial for developmental processes as well as a range of human diseases like imprinting disorders and cancer (Robertson, 2005). Methylation is maintained through mitosis and is also stable in free DNA of body fluids (e.g., plasma), making it a desirable biomarker. Detecting aberrant methylation is a promising approach for early diagnosis, classification and prognosis of cancer, and many studies have focused on this issue in the past years (Laird, 2003). However, these studies are spread throughout numerous journals. So far, two databases exist to assist researchers in assay design: MethDB (Amoreira *et al*, 2003) and MethPrimerDB (Pattyn *et al*, 2006). MethDB provides an overview of methylation evidence across more than 40 species; MethPrimerDB holds validated primer sequences and assay information on four major PCR methods used in CpG methylation analysis. A third database, called MethyCancer, will be published in 2008 and combines different major databases as well as CpC island sequencing data from the Chinese Cancer Genome/Epigenome Project (He *et al*, 2008).

## METHODS

### Annotation of the database entries

The diversity of data types and analytical methods combined with the fact that virtually all reports are paperbound made the assembly of MethCancerDB challenging. In total, we could allocate 4720 annotable entries from 4919 collected records, originating from 2240 different genes. Entries are gene-centred (instead of being sample- or study-centred). To cover all genes relevant to CpG methylation in cancer, we also included a list of census cancer genes (i.e., mutated genes causally implicated in oncogenesis) (Futreal *et al*, 2004) and imprinted genes as well as several review reports of methylated genes. Such sources are collectively marked as ‘secondary source’ to indicate that these are not original observations of aberrant methylation in cancer.

We implemented an annotation pipeline to extend the initial information of the entries. The gene identifier is sent to CGAP Batch Gene Finder (Strausberg, 2001) to obtain various levels of biological information, in particular, the official gene symbol (Eyre *et al*, 2006), the UniGeneID and the corresponding sequence IDs (e.g., RefSeqIDs). Then, all sequence IDs are annotated to their chromosomal position using the UCSC Genome Browser (Kent *et al*, 2002). CpG island information is crucial for tumour suppressor hypermethylation analysis. All CpG islands within 10 kb of the 5′ end of the sequence are located and their upstream (u) or downstream (d) position according to the 5′ ends is determined. Finally, sequences and general information of the CpG islands are transferred from UCSC to the MethCancerDB. Additionally, a ‘gene count’ sums up gene identifiers with the same EntrezGeneID (or the same UniGeneID for database entries without an EntrezGeneID). The gene count routine is executed for each new entry. If a gene occurs repeatedly in a single source (e.g., microarray profiling), the gene count increases only by one. The gene count does not increase when the entry is derived from a ‘secondary source’.

## RESULTS

### Web interface

The database section ‘explore’ includes the search functions of MethCancerDB. In our database, genes with their corresponding promoter CpG islands can be filtered, browsed, selected and downloaded. MethCancerDB supports searching for a combination of the following terms: source, gene symbol, cancer type, methylation status, study size, tissue type, method, sequence type, gene count and study type. Entries can be selected/deselected and downloaded by the ‘extract table’ function as tab-delimited files. The entire database can also be downloaded. To allow interaction of this database with other prominent resources, hyperlinks to PubMed, CGAP Gene info (including NCBI Gene information like Entrez Gene, UniGene, OMIM link, gene expression data, Gene Ontology terms, similar sequences and homologues) and to the three methylation-related databases, namely MethDB, MethPrimerDB and MethyCancer, are provided. Furthermore, the 5′UTR gene start site (±10 kb) of each SequenceID is hyperlinked to the UCSC browser and the chromosomal coordinates displayed in the database. In addition, all CpG islands from this region are listed with their relative downstream or upstream position to the SequenceID's 5′ end. Hyperlinks to meta-information and sequences of the CpG islands, as obtained by the pipeline, are provided.

Submission of novel entries can be done in the ‘submit’ section after registration and log-in. In the simplest case, a submission can contain as little information as a unique gene identifier (i.e., gene symbol, GenBank accession, Protein accession, UniProt accessions, UniGene ID or GeneID) and a PubMed ID (or alternatively an URL; all URLs are considered as ‘secondary source’). Further information is highly desired but not obligatory and can be selected from the respective menus. Selectable cancer types are derived from Jemal *et al* (2007). More complex submissions, which could include various sequence types, significance reports and personal comments, can be done by uploading a tabular data. A corresponding template and additional guidelines are provided in the ‘submit’ section. We then encourage submitters to provide information about the significance of the methylated gene for the investigated cancer phenotype (i.e., *P*-value, number of positive patients). New entries are automatically annotated and integrated into MethCancerDB using the pipeline described above. In the first release, only gene identifiers, but not the sequences, are annotated. In future, MethCancerDB will also store methylation information of intergenic sequences and not-annotated transcripts. An annotation pipeline for all sequences, including those already recorded in the database, is planned.

The section ‘match’ is an opportunity for researchers who wish to obtain methylation-relevant information for their genes but do not want to submit to the database. This might be the case, when a researcher obtained candidate genes for a malignancy from a large high-throughput study, like genome, transcriptome or proteome analyses, and is interested to know whether the resulting candidate genes are also aberrantly methylated. The list of candidate genes has to be uploaded as a txt file and information retrieval is done using the pipeline described above (also including the gene count). This information can be downloaded and is not saved to the database.

### Quality assurance and availability

MethCancerDB cannot be comprehensive without further submissions by the scientific community. Data in the initial release were extracted from PubMed by the authors; a senior biologist collected studies and another biologist integrated its entries into the database. A third biologist performed spot checks on the data. All changes to the database are monitored and dated. The database is updated after every new UniGene Build. Incorrect entries can be reported by e-mail using the entry's ID. All data of MethCancerDB are freely available.

## DISCUSSION

MethCancerDB provides a summary of pre-existing information regarding DNA methylation in various malignancies. A novelty of MethCancerDB is that its entries are based on entire studies instead of single patients/observations. In contrast to MethDB, MethPrimerDB and MethyCancer, in MethCancerDB, the experimental design (e.g., study size, type of cancer, method, etc.) is documented. Therefore, researchers can quickly overview all studies and their results for a specific clinical/biological scenario. Gene and CpG island information can be downloaded for the appropriate genes. Validated assays for a gene are indicated by links to the MethPrimerDB.

Annotating the genes to UniGene clusters makes it easier to compare the methylation status of different genes as opposed to comparing them on the basis of the original terms from PubMed articles. A candidate gene identified in any biological screen can be searched for a putative role in aberrant CpG methylation. This might be more important in the future as integrative approaches are gaining interest.

Completely independent from our database, bioinformaticians from the Chinese Cancer Genome/Epigenome Project developed a database, called MethyCancer, that integrates major databases dealing with either methylation, CpG island prediction or the cancer genome to a single display on the chromosomal level, called MethyView. MethyCancer will be published in January 2008 and is aimed at the beginning of DNA methylation research, whereas MethCancerDB collects the evidence from finished research and clinical studies. To strengthen the interaction between the databases, MethyView was connected to the MethCancerDB genes. In this way, the databases benefit from each other.

## References

[bib1] Amoreira C, Hindermann W, Grunau C (2003) An improved version of the DNA Methylation database (MethDB). Nucleic Acids Res 31: 75–77 12519951 10.1093/nar/gkg093 PMC165540

[bib2] Eyre TA, Ducluzeau F, Sneddon TP, Povey S, Bruford EA, Lush MJ (2006) The HUGO Gene Nomenclature Database, 2006 updates. Nucleic Acids Res 34: D319–D321 16381876 10.1093/nar/gkj147 PMC1347509

[bib3] Futreal PA, Coin L, Marshall M, Down T, Hubbard T, Wooster R, Rahman N, Stratton MR (2004) A census of human cancer genes. Nat Rev Cancer 4: 177–183 14993899 10.1038/nrc1299 PMC2665285

[bib4] He X, Chang S, Zhang J, Zhao Q, Xiang H, Kusonmano K, Yang L, Sun ZS, Yang H, Wang J (2008) MethyCancer: the database of human DNA methylation and cancer. Nucleic Acids Res 36: D836–D841 17890243 10.1093/nar/gkm730 PMC2238864

[bib5] Jemal A, Siegel R, Ward E, Murray T, Xu J, Thun MJ (2007) Cancer statistics, 2007. CA Cancer J Clin 57: 43–66 17237035 10.3322/canjclin.57.1.43

[bib6] Kent WJ, Sugnet CW, Furey TS, Roskin KM, Pringle TH, Zahler AM, Haussler D (2002) The human genome browser at UCSC. Genome Res 12: 996–1006 12045153 10.1101/gr.229102 PMC186604

[bib7] Kim TH, Barrera LO, Zheng M, Qu C, Singer MA, Richmond TA, Wu Y, Green RD, Ren B (2005) A high-resolution map of active promoters in the human genome. Nature 436: 876–880 15988478 10.1038/nature03877 PMC1895599

[bib8] Laird PW (2003) The power and the promise of DNA methylation markers. Nat Rev Cancer 3: 253–266 12671664 10.1038/nrc1045

[bib9] Pattyn F, Hoebeeck J, Robbrecht P, Michels E, De PA, Bottu G, Coornaert D, Herzog R, Speleman F, Vandesompele J (2006) methBLAST and methPrimerDB: web-tools for PCR based methylation analysis. BMC Bioinformatics 7: 496 17094804 10.1186/1471-2105-7-496 PMC1654196

[bib10] Robertson KD (2005) DNA methylation and human disease. Nat Rev Genet 6: 597–610 16136652 10.1038/nrg1655

[bib11] Strausberg RL (2001) The Cancer Genome Anatomy Project: new resources for reading the molecular signatures of cancer. J Pathol 195: 31–40 11568889 10.1002/1096-9896(200109)195:1<31::AID-PATH920>3.0.CO;2-W

